# Linking glycosphingolipid metabolism to disease-related changes in the plasma membrane proteome

**DOI:** 10.1042/BST20240315

**Published:** 2024-12-06

**Authors:** Holly Monkhouse, Janet E. Deane

**Affiliations:** Cambridge Institute for Medical Research, University of Cambridge, Cambridge CB2 0XY, U.K.

**Keywords:** glycosphingolipid, lysosomal storage disease, trafficking, vacuolar protein sorting proteins

## Abstract

Glycosphingolipids (GSLs) are vital components of the plasma membrane (PM), where they play crucial roles in cell function. GSLs form specialised membrane microdomains that organise lipids and proteins into functional platforms for cell adhesion and signalling. GSLs can also influence the function of membrane proteins and receptors, via direct protein-lipid interactions thereby affecting cell differentiation, proliferation, and apoptosis. Research into GSL-related diseases has primarily focussed on lysosomal storage disorders, where defective enzymes lead to the accumulation of GSLs within lysosomes, causing cellular dysfunction and disease. However, recent studies are uncovering the broader cellular impact of GSL imbalances including on a range of organelles and cellular compartments such as the mitochondria, endoplasmic reticulum and PM. In this review we describe the mechanisms by which GSL imbalances can influence the PM protein composition and explore examples of the changes that have been observed in the PM proteome upon GSL metabolic disruption. Identifying and understanding these changes to the PM protein composition will enable a more complete understanding of lysosomal storage diseases and provide new insights into the pathogenesis of other GSL-related diseases, including cancer and neurodegenerative disorders.

## Introduction

**Figure 1.**
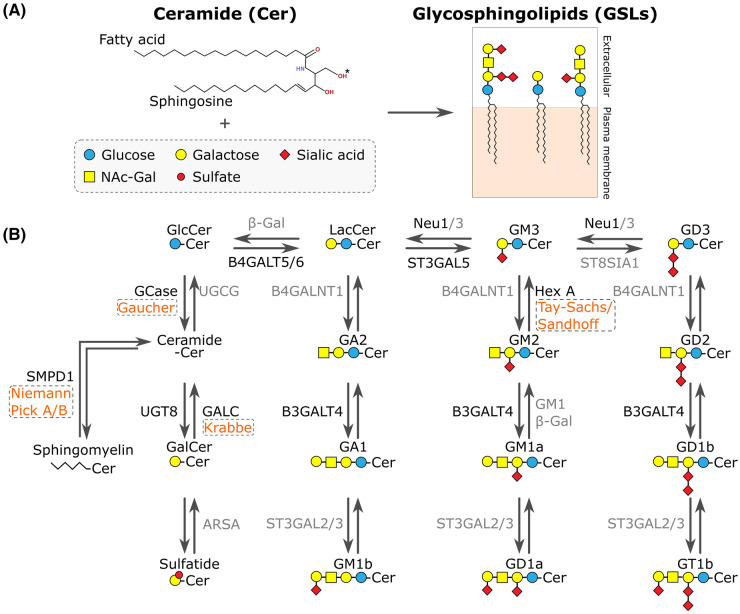
Glycosphingolipid (GSL) metabolism and sphingolipidoses. (**A**) The GSLs are formed by the addition of the sugar moieties glucose (blue circle), galactose (yellow circle), sialic acid (red diamond), *N*-acetyl-galactosamine (NAc-Gal, yellow square), and sulfate (red dot), to a ceramide backbone. (**B**) Simplified schematic of GSL metabolism and disease pathways. Lysosomal Storage Disorders (LSDs) caused by mutations in GSL degradative enzymes are in orange and boxed. Enzymes highlighted in black are mentioned in the main text.

Sphingolipids are bioactive lipids possessing a ceramide backbone composed of sphingosine and a fatty acid. An important subclass of sphingolipids, known as glycosphingolipids (GSLs), have headgroups composed of glycan moieties ranging from a single glucose or galactose to highly complex branched glycans (Figure 1A) [1]. Complex GSLs containing one or more sialic acid residue are called gangliosides [2]. GSLs are enriched in the outer leaflet of the plasma membrane (PM) where they play crucial roles in cell signalling, cell adhesion and cell fate via interactions with PM proteins [3–5]. During development GSL abundances change with different cell types displaying distinct repertoires of GSL species on their surface, helping to define cellular identity [5]. Therefore, maintaining the correct abundance of different GSLs is important for development and maintaining cellular function.

GSLs are synthesised in the endoplasmic reticulum (ER)/Golgi and are degraded in the endolysosomal system. This process involves a broad array of different synthetic and degradative enzymes that process these lipids in a stepwise manner (Figure 1B) [1]. GSL metabolism also requires several, specific lipid-binding and lipid-transport molecules. For detailed information on ganglioside structure and biosynthesis, see Ref. [6]. The structure and biophysical properties of GSLs support *cis-*interactions with other sphingolipids, cholesterol, and membrane proteins to form specialised membrane microdomains (also referred to as lipid rafts) [7]. Clustering of GSLs also facilitates *trans*-interactions with molecules located in the extracellular matrix or PM of adjacent/nearby cells driving cell adhesion [8].

GSL imbalances are associated with a range of diseases. These include diseases where GSL degradation in the endolysosomal system is compromised due to loss of hydrolytic enzymes, lipid transporters or other cofactors, leading to accumulation of undigested material in these compartments [1]. These diseases are part of the large family of lysosomal storage disorders (LSDs). GSL imbalances can also be caused by loss of activity of the enzymes that synthesise GSLs, leading to rare forms of hereditary spastic paraplegia and epilepsy [9,10]. GSL abundance changes are also associated with more common diseases including many types of cancer from childhood neuroblastoma to ovarian cancer (reviewed in [11,12]), as well as late-onset neurodegenerative diseases including Parkinson's and Alzheimer's disease (reviewed in [13,14]).

Previous reviews have highlighted the potential for accumulating lipids in the lysosome to ‘spill over’ into other organelles altering their architecture and perturbing cellular functions beyond the lysosome [15,16]. Leakage of lysosomal contents, altered lipid trafficking and fusion of lysosomes with the PM have been proposed as mechanisms driving the neurotoxicity seen in LSDs. These proposals are supported by growing evidence that in LSDs, the accumulation of undigested GSLs does have consequential effects for other membranes and organelles including the ER, mitochondria and PM. These effects are complex, involving multiple membrane components, with the molecular mechanisms only beginning to be unravelled. These effects on different cellular compartments have primarily been characterised for LSDs but have also been proposed to play a role in other, less well studied GSL-related diseases such as those involving GSL biosynthetic enzymes [17]. Due to the bioactive nature of GSLs and their crucial functions at the PM in cell signalling and cell fate, it is likely that changes at the PM will have important phenotypic consequences that directly influence disease pathology. In this mini-review we will focus on the mechanisms by which disrupted GSL levels can alter the PM proteome and explore the current evidence for these changes in GSL-related diseases.

## Lysosomal exocytosis

**Figure 2.**
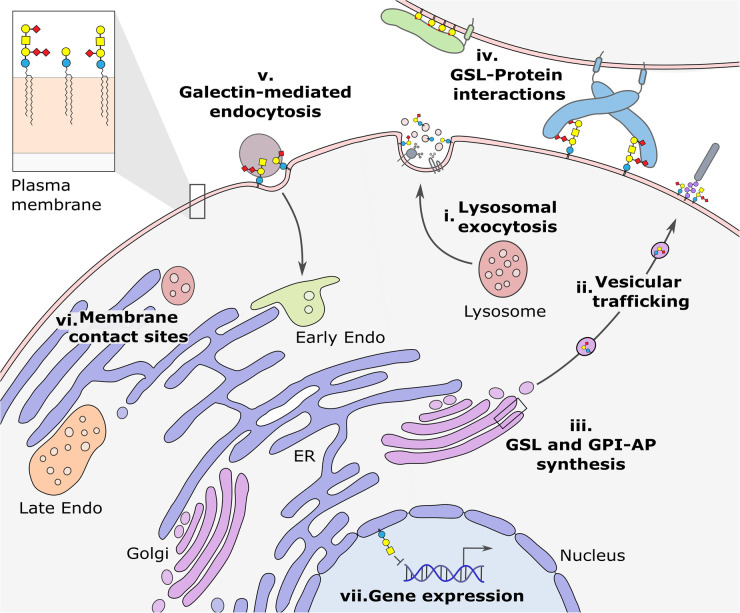
Overview of the molecular mechanisms by which GSLs can alter protein abundance at the plasma membrane (PM). Each of the mechanisms described in the text are illustrated in the context of a cell. The PM composition of a cell can be altered when: (**i**) lysosomal exocytosis delivers endolysosomal components to the PM; (**ii**) vesicular trafficking and sorting is altered in the Golgi; (**iii**) GPI-anchored protein delivery also involves GSL-dependent sorting in the Golgi; (**iv**) direct GSL-protein interactions can stabilise proteins at the PM while; (**v**) galectin-mediated endocytosis can mediate internalisation of PM proteins; (**vi**) membrane contact site dynamics may be responsible for altering the lipid and protein composition of the PM; and (**vii**) gene expression changes can be triggered by GSL imbalances or GSLs present in the nuclear membrane. Abbreviations: Endo, endosome; ER, endoplasmic reticulum.

In diseases involving the lysosome, such as LSDs, the changes at the PM could be driven by lysosomal exocytosis [18]. This is a calcium-regulated process whereby lysosomes dock at the PM and fuse their limiting membrane with the PM to release their lumenal contents into the extracellular space (Figure 2i) [19]. Lysosomal exocytosis plays an important role in PM repair [19] and in the regulated secretion of lysosomal contents in specialised cells [20]. However, in diseases where lysosomes accumulate unprocessed cellular debris, increased exocytosis can be triggered, resulting in changes to the composition of the PM and release of lysosomal contents into the extracellular space [19,21]. The accumulation of cholesterol and sphingolipids in the lumen of lysosomes has been directly implicated in influencing lysosomal exocytosis [18]. Sphingolipids are present on the luminal faces of both the limiting membrane and intralumenal vesicles, and changes in their abundance in these membranes may influence the activity of membrane proteins involved in fusion, fission and exocytosis, such as SNARE complexes and TRPML1 [18,22,23].

The role of lysosomal exocytosis in altering the PM composition was explored in a model for lysosomal storage using fibroblasts loaded with sucrose [24]. These cells displayed increased lysosomal biogenesis and lysosomal exocytosis. This resulted in changes to the PM GSL composition and an increase in the abundance of the lysosomal membrane protein LAMP-1 at the PM. The activity of lysosomal enzymes was also increased at the PM and in the extracellular medium further supporting that sucrose-loading of lysosomes triggered fusion with the PM. This work supports that functional impairment of lysosomes due to substrate storage drives increased lysosomal exocytosis resulting in changes to the GSL and protein composition of the PM.

A recent study has looked at changes to the PM proteome in two new iPSC neuron-based models of the LSDs, Tay-Sachs and Sandhoff diseases [25]. In both these diseases GM2 accumulates due to defects in the subunits of the β-*N*-acetylhexosaminidase A (Hex A) enzyme (Figure 1B). In addition to substantial lysosomal accumulation of unprocessed lipids, GM2 was also shown to accumulate at the PM, supporting that the impact of lysosomal accumulation of GSLs is not restricted to the endolysosomal compartments. Using surface biotinylation and streptavidin-mediated enrichment, significant changes were also identified in the PM proteome. Amongst the proteins increased in the disease models were several lysosomal proteins including SCARB2, TSPAN3, ACP2, NPC1 and LAMTOR1. This provides strong evidence that lysosomal exocytosis is a pathological outcome of GM2 accumulation and is driving protein abundance changes at the PM.

Lysosomal exocytosis has been implicated as the mechanism driving pathology in a mouse model of sialidosis caused by loss of lysosomal sialidase NEU1 (Figure 1B) [26,27]. In this disease, excessive sialylation of amyloid precursor protein (APP) leads to its accumulation in the lysosome and an increase in lysosomal exocytosis resulting in increased abundance of lysosomal proteins, such as LAMP1, at the PM as well as excessive release of Aβ peptides. Furthermore, in neu1^−/−^ mice, this process also results in the release of serine proteases that inactivate extracellular serpins, cause premature degradation of VCAM-1, and drive loss of bone marrow retention [28] highlighting the impact of lysosomal dysfunction on the extracellular matrix and bystander cells.

## Vesicular trafficking and recycling

GSL imbalances may also influence the PM protein composition via secretory trafficking from the ER-Golgi network to the PM (Figure 2ii). The glycolipid transfer protein (GLTP) is a regulator of GSL homeostasis and, via its interactions with VAP-A, is involved in vesicular trafficking [29]. Knockout of GLTP in HeLa cells altered the GSL composition of cells and reduced the trafficking of a reporter construct, VSVG-GFP, to the PM. Although direct changes to the abundance of endogenous PM proteins was not tested, the consequence of GLTP loss in cells was increased cellular adhesion and motility, supporting a link between altered GSL abundance and disrupted PM protein composition.

Membrane trafficking, endocytosis and vesicle secretion have been identified as defective in several GSL-related LSDs providing additional mechanisms that may influence the composition of the PM, particularly at the synapse. In neurons from the naturally-occurring Twitcher mouse model of the LSD Krabbe disease, the transport of endocytic and synaptic vesicles was impaired potentially due to the impact of cellular psychosine accumulation [30,31]. Krabbe disease is caused by mutations in the enzyme galactocerebrosidase (GALC) or its lipid-binding cofactor Saposin A, which prevents the degradation of galactosylceramide (GalCer) to ceramide (Figure 1B) [32]. As a result of GalCer accumulation, overdependence on an alternative degradative pathway leads to psychosine production and accumulation [33]. In Krabbe disease, psychosine has also been implicated in the disruption of membrane microdomains, membrane destabilisation and shedding of microvesicles from the PM [34]. A related LSD, Gaucher disease, is caused by mutations in the lysosomal enzyme glucocerebrosidase (GCase) leading to the accumulation of glucosylceramide (GlcCer) (Figure 1B) [35]. This GSL accumulation causes altered PM fluidity and increased raft size resulting in reduced endocytosis of the non-raft protein transferrin receptor [36]. The LSD Niemann-Pick disease type C1 (NPC1) is caused by the loss of function of the lipid transporters NPC1 or NPC2 leading to the accumulation of several GSLs in addition to cholesterol [37]. In hippocampal neuronal cultures of NPC1 mice, drastic defects were identified in evoked vesicle exocytosis due to a delay in recycling of exocytosed vesicles with new fusion-competent vesicles [38]. As the rate of synaptic vesicle recycling directly controls neurotransmitter release, deficits in these endocytic and exocytic processes can affect synaptic function, composition and signalling strength.

Proteomic analysis of different models of GSL metabolic diseases have identified specific changes to the abundance of synaptic proteins at the PM. In models of GM3 synthase deficiency, the PM abundance of several proteins were altered including the synaptic proteins NRP1, GPC4 and several members of the neuroligin (NLGN) family of proteins [39]. NLGNs are important for synaptogenesis and synapse remodelling but also for synaptic vesicle clustering suggesting a link between GSL synthesis, NLGN function and synaptic vesicle trafficking [40–42]. Several synaptic proteins were also altered in their PM abundance in neuronal models of GM2 gangliosidoses [25]. One of these was synaptotagmin 1 (SYT1), a crucial protein in synaptic vesicle fusion and recycling [43]. Interestingly, SYT1 directly binds membrane lipids including the GSL GT1b [44]. The observation of increased SYT1 at the PM in these disease models, suggests that vesicular trafficking and recycling defects are not only due to general disruption of membrane recycling or fluidity, but could be driven by direct protein-GSL interactions.

## GPI-anchored proteins

The GPI anchor is a glycolipid-based post-translational modification made to several cell-surface proteins to form GPI-anchored proteins (GPI-AP, Figure 2iii). GSLs interact with GPI-APs to form dynamic membrane microdomains that form at the trans-Golgi network and are transported to the PM [45]. The sorting and trafficking of GPI-APs to the PM is complex involving multiple factors including modifications to the GPI anchor itself, oligomerisation of GPI-APs and N-linked glycosylation [46–49]. Of specific relevance to this review, is that inhibition of sphingolipid biosynthesis and/or removal of cholesterol impairs the trafficking of GPI-APs to the apical membrane demonstrating that their trafficking is, in part, dependent upon their inclusion into GSL-rich microdomains [49,50]. Although interactions with GSLs may not be the primary mechanism of GPI-AP sorting and trafficking, GSL dysregulation may play a role in altering the PM abundances of GPI-APs [51].

Interactions between GPI-APs and GSLs may be transient and evidence for specific interactions in the Golgi remains unclear. However, it is worth noting that in several models of diseases involving altered GSL metabolism, specific GPI-APs have been identified that are altered in their PM abundance [25,39,52]. For example, glypican (GPC) proteins are differently affected in these models with GPC6 being decreased at the PM in a Krabbe disease model [52], while in GM2 gangliosidoses models, GPC4 is decreased [25] and in GM3 synthase deficient cells, GPC4 accumulates at the PM [39]. Similarly, cadherins and contactin proteins, both GPI-AP families are differentially affected in these models. The altered PM abundance of different GPI-APs in these models with disrupted GSL metabolism suggests that the specific GSLs that are accumulating or lost in these diseases may influence the GPI-AP repertoire that is delivered to the PM.

Although specific interactions have been difficult to observe, GPI-APs and GSLs likely function together to mediate sorting in the Golgi and trafficking to the PM [53]. Recently, B3GALT4 (Figure 1B), which mediates the biosynthesis of the GSLs GA1, GM1a, and GD1b, was shown to also function in GPI-anchor formation by transferring galactose to the *N*-acetylgalactosamine sidechain of GPI [54]. Furthermore, B3GALT4 requires lactosylceramide (LacCer) to efficiently carry out this reaction, demonstrating complex functional relationships between GPI-anchored proteins and GSLs in the Golgi [55]. Therefore, the abundance of GSLs and the enzymes that synthesise them may directly influence the GPI-AP composition of the PM.

## Direct GSL-protein interactions

Several studies have demonstrated direct interactions between GSLs and membrane proteins (reviewed in [56,57]). However, there are relatively few studies linking these interactions to altered protein abundance at the PM (Figure 2iv). Recently, new clickable photoaffinity ganglioside probes have been developed and used to provide insights into the ganglioside interactome [58]. This study compared the proteins that are enriched following cross-linking to GSLs with tags attached to the ceramide tail or to the glycan headgroups. Some of the identified protein targets were common across the GSL species tested suggesting they may represent non-specific co-clustering of PM proteins with GSLs. However, the identification of non-overlapping proteins using tags attached to different GSLs suggests that some of the identified interactions represent specific GSL-protein interactions. This study identified both expected, including GPI-APs, and new potential protein interactions with GM3, GM1 and GD1a in two human cell lines: A-431 (epidermoid carcinoma) and SH-SY5Y (neuroblastoma) cells. Comparison of the proteins identified in this study with those that are altered in GSL metabolic diseases may contribute to a better understanding of whether direct protein-GSL interactions can alter the PM proteome.

In iPSC-derived neural crest cells with mutant ST3GAL5, a ganglioside biosynthetic enzyme (Figure 1B), cell surface glycoproteins were labelled with biotin using Selective Exo-Enzymatic Labelling. The biotinylated proteins were affinity-enriched and analysed by SDS–PAGE and mass spectrometry [39]. Almost half of the proteins identified as altered in their cell surface abundance were also identified in the ganglioside interactome study [39,58]. This includes cell adhesion molecules (such as ITGA3/6, NLGN2, EPHA2), ion transporters (such as ATP1A2, SLC6A8, CACNA2D2), Wnt-associated proteins (ROR1 and RECK) and NOTCH-associated proteins (JAG1 and NOTCH2) as well as other proteins including LAMP1. The loss of ST3GAL5 function would block the synthesis of several GSLs, including GM3, GM1 and GD1a suggesting that the overlapping list of proteins in these studies highlights a potential role for direct GSL-protein interactions in altering PM protein abundance. However, in this study no mechanism for how these changes occur was proposed.

Interestingly, many of the proteins identified as potential GSL binders were also identified as changed in their abundance at the cell surface in new models of the LSD GM2 gangliosidosis [25]. These changes included both lysosomal (TSPAN3, CD63 and LAMP1) and non-lysosomal PM proteins (TTYH3, NTRK2/3, ROR1, EGFR amongst others). The overlapping list of proteins suggests that the excess GM2 in these disease models may be disrupting normal GSL-protein interactions allowing the formation of non-native complexes that interfere with normal trafficking. GM2 is not normally present at high abundance in neurons and these altered interactions may by driving the lysosomal exocytosis and synaptic recycling defects observed in this study. The correlations between these datasets may represent a link between direct protein-GSL interactions and PM abundance changes but further research is required to test this directly.

In support of the potential link between direct GSL interactions and PM protein abundance there are also overlapping proteins identified as GSL-binders in cell lines with disruption of GalCer metabolism. The GalCer synthetic (UGT8) and degradative (GALC) enzymes were knocked out (KO) in oligodendrocyte-like cells leading to significant changes in the PM proteome [52]. Within the list of highly confident hits were several proteins that were also identified via the photoaffinity probe experiments or are closely-related proteins including ROR2, ATP2A1, NECTIN-1, SEMA6D, NLGN1, PTPN13 as well as neurofascin (NFASC). In this study, NFASC was significantly and reciprocally altered between these two cell lines, having increased abundance in the GALC KO and decreased abundance in the UGT8 KO. This change was consistent with a direct interaction between a galactose-containing GSL and NFASC. Previous work supports that NFASC localisation or stability at the paranode, a region of tight membrane contact between the axon and myelin sheath, depends upon the sulfated GalCer, sulfatide [59,60]. In this study, using *in vitro* binding assays, the myelin-specific isoform of NFASC (NF155) was shown to directly and specifically bind multiple sulfatide molecules and that this drives extensive *cis* membrane interactions, potentially orienting the NF155 extracellular domain such that it lies flat against the membrane within the narrow myelin-axon space [52]. This extensive interface between NF155 and the membrane may contribute to its stability at the PM and explain the abundance changes upon GSL disruption.

In addition to *cis* interactions between GSLs and membrane proteins, *trans* interactions are also important for maintaining proteins at the PMs of axon-myelin junctions. In the absence of GSL synthesis the myelin associated glycoprotein (MAG), a type 1 membrane protein, is lost from the internode [59]. MAG is known to interact with myelin membrane microdomains containing sulfatide, and the loss of both sulfatide and complex gangliosides from the myelin and axon membranes respectively exacerbates MAG mislocalisation and subsequent myelin disorganisation [59,61]. MAG specifically interacts with GSLs in the axon membrane that contain the trisaccharide Neu5Ac-α2,3-Gal-β1,3-GalNAc [62] present in GT1b, GD1a, and GM1b. The structure of the full-length MAG extracellular domain bound to this trisaccharide suggests that it forms a homodimer and interacts with axonal membrane-embedded GSLs in *trans* [63]. These data support that protein-GSL interactions between membranes also contribute to PM protein localisation and stability.

## Galectin-mediated endocytosis

GSL-protein interactions are not just important in neuronal cells. Direct interactions between GSLs and cellular lectins, such as galectins, play an essential role in clathrin-independent endocytosis [64] (Figure 2v). This assembly of tubular endocytic pits via membrane bending is a GSL-dependent process, referred to as the glycolipid-lectin hypothesis [65,66]. This mechanism was recently shown to be required for the transport of the PM protein lactotransferrin from the apical to the basolateral membrane of enterocytes [67]. It remains to be seen whether galectin-mediated endocytosis is disrupted, and consequently the PM proteome altered, in diseases where GSL metabolism is affected.

## Membrane contact sites

Recently, GSL imbalances associated with neurodegenerative diseases have been implicated in the disruption of signalling at membrane contact sites (MCSs) [68]. MCSs are formed when membrane microdomains within organelles or the PM come into close proximity without fusing (Figure 2vi). Tethering of these membranes, via specific lipid and protein interactions, facilitates the transport of lipids across the MCS and the exchange of metabolites such as calcium [68]. When GSLs accumulate abnormally in intracellular membranes or the PM, this can disrupt the topology of MCSs disrupting their function. Dysfunctional MCSs have been identified in the GSL-related LSDs GM1-gangliosidosis, NPC1 and Gaucher disease [69–72]. These defects have been attributed to the accumulation of GSLs and/or cholesterol at MCSs and the inappropriate transfer of these lipids into different organelle membranes. Whether these disruptions result in changes in the protein composition of the MCS or organelle more generally remains to be elucidated.

## GSL-mediated changes to gene expression

Changes to the PM proteome can also occur via GSL-mediated effects on gene expression including altering the expression levels of specific PM adhesion molecules (Figure 2vii). Silencing of the lactosylceramide synthase gene (B4GALT5/6) (Figure 1B) in astrocytes attenuates expression of the adhesion molecules ICAM-1 and VCAM-1 [73]. Expression of the GSL biosynthetic gene sulfotransferase, that produces lactosylsulfatide SM3, reduces the expression of β1 integrin resulting in reduced cell adhesion [74]. GSLs can also influence epigenetic pathways during neuronal differentiation, a process involving a distinct change in GSL profile of these cells [75]. The globo-series GSLs repress AUTS2, the epigenetic regulator of neuronal gene expression. AUTS2 then activates the promoter of the first and rate-limiting ganglioside-producing enzyme GM3 synthase (ST3GAL5) (Figure 1B), triggering the synthesis of the complex gangliosides. This thereby links GSL reprogramming to neural gene expression during differentiation. Although there have not been many studies looking at general changes to the transcriptome of cells with altered GSL metabolism, a recent study showed that knockout of the lipid transporter GLTP resulted in altered GSL levels and significant gene expression changes, including the up-regulation of several genes involved in the endomembrane system [29].

## Conclusion

This mini-review provides several examples of how membrane trafficking and vesicle recycling may be disrupted by changes in GSL metabolism and GSL abundance. Beyond general membrane fluidity or dynamics defects, there are several examples of direct and specific GSL-protein interactions that may contribute to the dysfunction in these processes leading to changes in the PM abundance of membrane proteins. The mechanisms highlighted here are not mutually exclusive and are likely to be intimately interlinked. Some of the highlighted mechanisms, particularly altered gene expression and disrupted PM contact sites, require further study to understand the extent of their impact on the PM proteome.

Several of the studies described here involved monitoring specific PM proteins of interest, such as transferrin receptor, APP or LAMP-1, rather than profiling the entire PM proteome for a more general insight into PM changes. Although there is substantial circumstantial evidence for such changes in LSDs, due to significant alterations to synaptic signalling, cell adhesion and immune responses, there are relatively few studies exploring the PM proteome using unbiased approaches that could capture a fuller picture of the impact of GSL changes in these diseases. Exploration of the broader consequences of GSL imbalances on the PM proteome and subsequent impact on cell adhesion and cell signalling is an exciting and growing area for future research.

## Perspectives

- GSLs play crucial roles in cell adhesion, signalling, and determining cell fate, with imbalances leading to a range of devastating diseases from cancer to neurodegeneration.
- Research into GSL metabolic diseases has primarily focussed on lysosomal dysfunction, but recent work is revealing that GSL imbalances have more widespread effects, impacting the composition and functions of other cellular compartments.
- To develop a more complete understanding of GSL-related diseases, future research must include analysis of the impact GSL imbalance has beyond lysosomal dysfunction, particularly changes at the PM, where GSLs carry out their primary functions.

## Open Access

Open access for this article was enabled by the participation of University of Cambridge in an all-inclusive *Read & Publish* agreement with Portland Press and the Biochemical Society under a transformative agreement with JISC.

## Abbreviations

APP: amyloid precursor protein
ER: endoplasmic reticulum
GALC: galactocerebrosidase
GLTP: glycolipid transfer protein
GSL: glycosphingolipid
KO: knocked out
LSD: lysosomal storage disorder
MAG: myelin associated glycoprotein
MCS: membrane contact site
PM: plasma membrane
